# Single-Cell Comparison of Small Intestinal Neuroendocrine Tumors and Enterochromaffin Cells from Two Patients

**DOI:** 10.3390/cancers18030435

**Published:** 2026-01-29

**Authors:** Fredrik Axling, Elham Barazeghi, Per Hellman, Olov Norlén, Samuel Backman, Peter Stålberg

**Affiliations:** Department of Surgical Sciences, Uppsala University, SE-751 85 Uppsala, Sweden; elham.barazeghi@uu.se (E.B.); per.hellman@uu.se (P.H.); olov.norlen@uu.se (O.N.); samuel.backman@uu.se (S.B.)

**Keywords:** neuroendocrine tumors, SI-NET, enterochromaffin cell, scRNA-seq, transcriptomics

## Abstract

Background: Several studies have attempted to identify the initiating drivers of small intestinal neuroendocrine tumor (SI-NET) development and the molecular mechanisms underlying their progression and metastatic spread. Previous gene expression studies have used bulk microarrays or RNA sequencing to compare tumor tissue with normal intestinal mucosa. However, the intestine comprises multiple distinct cell types, and bulk analyses are limited by this cellular heterogeneity, which can confound tumor-specific signals. Methods: We performed single-cell RNA sequencing on primary SI-NETs and paired normal mucosa from two patients to directly compare tumor cells with their cells of origin, the enterochromaffin (EC) cells. To minimize type I errors, we applied a two-step validation strategy by overlapping differentially expressed genes with an external single-cell dataset and cross-referencing candidate genes for enteroendocrine expression in the Human Protein Atlas. Results: For further distinction and characterization, ECs were subdivided into serotonergic and non-serotonergic clusters. This analysis revealed that the SI-NET cells are transcriptionally more similar to serotonergic ECs, consistent with serum metabolite profiles derived from clinical parameters. Our analyses uncovered a loss-of-expression program characterized by regulators of epithelial differentiation and in parallel, a gain-of-expression program displayed neuronal signaling gene induction, implicating functional reprogramming toward neuronal-like properties. Together, these specific losses and gains suggest that our patient-derived SI-NETs undergo adaptation through both loss of enteroendocrine functions and acquisition of neurobiological-promoting signaling pathways. Conclusions: These findings nominate candidate drivers for further functional validation and highlight potential therapeutic strategies in our patient cohort, including restoring suppressed Notch signaling and targeting aberrant neuronal signaling networks. However, even with a two-step validation procedure, the modest cohort size limits statistical power and generalizability, particularly for the proposed association to a serotonergic phenotype. Larger, multi-patient single-cell studies are required to confirm these mechanisms and establish their clinical relevance.

## Simple Summary

Small intestinal neuroendocrine tumors develop from hormone-producing cells in the gut, yet the changes that cause these cells to become cancerous are still not well understood. Most earlier studies analyzed whole tissue samples, which makes it difficult to separate signals coming from normal cells from those of hormone-producing normal cells. In this study, we examined tumors at the level of individual cells and compared them directly with their corresponding normal hormone-producing counterparts. This allowed us to identify gene activity changes that are specific to the tumor cells. We discovered that the tumor cells lose traits that are typical of endocrine intestinal cells and gain traits that are more commonly associated with nerve cells. These shifts suggest that the tumor cells undergo functional reprogramming as they develop. Our findings offer new insight into the tumor biology of our patients and may help us guide future research into targeted treatment strategies.

## 1. Introduction

Small intestinal neuroendocrine tumors (SI-NETs), which are believed to originate from enteroendocrine enterochromaffin (EC) cells of the gastrointestinal tract, are typically slow-growing and small in size, with an annual incidence of approximately 1 per 100,000 individuals. Most patients are diagnosed at stage IV, with distant metastases [1,2,3,4].

Studies investigating epigenetic and genetic alterations in SI-NETs remain limited. Extensive DNA analyses using short-read sequencing and array-based methods have revealed a relatively stable tumor genome, frequently characterized by loss of chromosome 18 but lacking recurrent smaller mutations [5], except for mutations in CDKN1B, which occur in approximately 8% of cases [6,7].

Several studies [8] have attempted to determine the pathological origin of SI-NETs and elucidate the molecular mechanisms underlying their progression and metastatic spread. For this purpose, normal intestinal mucosa has commonly been used as a control in gene expression studies employing bulk microarrays or RNA sequencing. Although bulk analyses are a mainstay of molecular biology research, they are limited by tissue heterogeneity, as gene expression measurements represent an average across multiple cell types [9].

Single-cell RNA sequencing (scRNA-seq) is particularly valuable for SI-NETs, which are thought to arise from a rare EC cell population within the intestinal epithelium. The intestinal epithelium itself comprises several distinct cell types [10,11], not solely the EC cells. Consequently, bulk RNA sequencing produces results that may not be specific to the SI-NET phenotype but instead reflect broader differences, as between SI-NET cells and healthy enterocytes. The scarcity of appropriate control samples may therefore contribute to the limited translational progress in identifying effective targeted therapies for SI-NETs.

To address this limitation, we applied scRNA-seq to isolate EC cells from resected normal intestinal mucosa using specific cell markers and compared their transcriptomic profiles with those of surgically resected SI-NET tumors. In the present study, we aimed to compare the transcriptomes of SI-NET cells and normal EC cells to identify driver genes and gene modules with specific aberrant expression.

## 2. Materials and Methods

### 2.1. Patient Samples

Tumor tissue and adjacent normal intestinal mucosa were collected from two patients undergoing surgical resection for distal ileum SI-NETs. Preoperative biomarkers included serum chromogranin A (S-CgA) and serum 5-hydroxyindoleacetic acid (S-5HIAA). Ki-67 indices were assessed by immunohistochemistry.

Patient 1 was a 70-year-old female with a grade 1, stage III SI-NET (pT3N2M0), elevated S-CgA at 3.5× the upper reference limit (URL), S-5HIAA at 2× the URL, and a Ki-67 index of 2.7%. Patient 2 was a 65-year-old male with a grade 2, stage IV SI-NET (pT4N2M1), S-CgA at the URL, S-5HIAA elevated at 4.8× the URL, and a Ki-67 index of 3.0%.

### 2.2. Sample Preparation, Library Construction, and scRNA Sequencing

During the gross pathological examination of surgical specimens from two patients with SI-NETs, representative pieces of tumor tissue and adjacent normal bowel mucosa were collected for analysis. The tissue was minced, washed with Ham’s F-10 medium (Sigma N-2147; MilliporeSigma, Burlington, MA, USA), and centrifuged to separate cells from the medium. Cells were then transferred to a collagenase solution containing 1 mg/mL collagenase (C-9263; MilliporeSigma, Burlington, MA, USA), 0.05 mg/mL DNase I (DN-25; MilliporeSigma, Burlington, MA, USA), 1.5% (*w*/*v*) bovine serum albumin (BSA), and 0.95 mM CaCl_2_, prepared in Nutrient Mixture F-10 Ham (N-2147; MilliporeSigma, Burlington, MA, USA), pH 7.4, and incubated at 37 °C for 2 h.

During incubation, the cell suspension was intermittently dispersed by gentle pipetting and placed on a shaking table at 200 rpm to facilitate tissue dissociation. After incubation, the suspension was filtered through sterile nylon mesh with a 125 µm pore size (NY125 HD; Sefar Group, Thal, Switzerland) to separate dispersed cells and small tissue fragments from larger pieces. Cells were then centrifuged and washed with EGTA buffer (Thermo Fisher Scientific, Waltham, MA, USA) containing 142 mM NaCl, 6.7 mM KCl, 10 mM HEPES, and 1 mM EGTA, pH 7.4. The cell pellet was resuspended in a 25% Percoll gradient solution (MilliporeSigma) prepared from 100% Percoll diluted to 25% (*v*/*v*) with 1× PBS, pH 7.4, and sterile-filtered to obtain a single-cell suspension.

Single-cell libraries were prepared using the Chromium Single Cell 3′ Reagent Kit v3 (10x Genomics, Pleasanton, CA, USA) according to the manufacturer’s instructions and sequenced on a NovaSeq 6000 SP flow cell (Illumina, San Diego, CA, USA). Raw sequencing reads were processed with Cell Ranger v7.2.0 (10x Genomics) to demultiplex samples, assign cell barcodes, and generate gene expression count matrices.

### 2.3. External scRNA-Seq Datasets

Published single-cell datasets [12,13] were obtained for external validation. EC and SI-NET cells were extracted following the same processing pipeline as the internal dataset. Gene expression patterns were cross-referenced with the Human Protein Atlas single-cell transcriptomics dataset [14] to confirm enteroendocrine expression.

### 2.4. Bioinformatics Processing and Data Analysis

Raw sequencing reads from internal and external scRNA-seq datasets were processed using Cell Ranger v7.2.0 (10x Genomics, Pleasanton, CA, USA) with the 2020-A Human GRCh38 transcriptome reference (GENCODE v32/Ensembl 98) to generate count matrices, which were analyzed using Seurat v5.3.0 [15] in R v4.4.3.

Quality control for internal samples involved removing cells with fewer than 200 or more than 7000 features or with >40% mitochondrial gene expression to exclude low-quality cells. External datasets were filtered according to their respective publications: primary SI-NET samples [12] excluded cells with fewer than 250 or more than 5000 features or >10% mitochondrial content, and small intestine samples [13] excluded cells with fewer than 250 or more than 5000 features or >50% mitochondrial content. For the reanalysis of the Wang dataset, to improve validation and quality control, we applied a more conservative cutoff at >30% mitochondrial gene expression, as the original 50% threshold was too permissive relative to healthy cells and the general cell population in the dataset. Cells showing high expression of stress-associated genes (*HSPA1A*, *HSPA1B*, *HSP90AA1*, *HSPB1*, *DNAJB1*) above the 98th percentile were considered distressed and removed [16,17,18,19].

To minimize batch effects between samples, datasets were integrated using Seurat’s reciprocal Principal Component Analysis (RPCA)-based workflow. For each condition (tumor and normal ileum), filtered and normalized Seurat objects were first processed to identify 2000 variable features, scaled, and subjected to principal component analysis. Integration anchors were calculated using FindIntegrationAnchors with RPCA reduction, and datasets were merged using IntegrateData to generate batch-corrected expression matrices. The integrated assays were then scaled and visualized using Principal Component Analysis (PCA) and Uniform Manifold Approximation and Projection (UMAP), followed by unsupervised clustering (FindNeighbors, FindClusters) to identify biologically distinct populations while minimizing technical variance.

Differential gene expression and group-wise distributional differences between cell clusters and cells were assessed using the Mann–Whitney U test, with Bonferroni correction applied for multiple testing (*p*.adj ≤ 0.05), unless stated otherwise. EC and SI-NET clusters were identified in both tumor and normal mucosa using unsupervised clustering guided by canonical EC markers: Chromogranin A (*CHGA*), Chromogranin B (*CHGB*), Secretagogin (*SCGN*), Neuronal Differentiation 1 (*NEUROD1*), Solute Carrier Family 18 Member 1 (*SLC18A1*), and Adhesion G Protein–Coupled Receptor G4 (*ADGRG4*) [20,21,22,23,24,25]. Identified EC clusters from tumor and normal samples were extracted and merged into a single Seurat object containing both tumor and normal EC cells. The merged dataset was scaled and reduced by PCA and UMAP to remove residual batch variance and enable direct comparison. The internal discovery dataset was analyzed in parallel with the similarly processed external sample dataset, which served as an independent validation cohort. Markers for all other cell types are listed in Table S1. Aneuploidy and copy number variations (CNVs) in the cells were analyzed using CopyKAT v1.1.0 [26] and inferCNV v1.26.0.

EC cell and SI-NET subtyping was performed using a panel of subtype-specific canonical markers: Tryptophan Hydroxylase 1 (*TPH1*), Dopa Decarboxylase (*DDC*), *SLC18A1*, *CHGA*, *CHGB*, LIM Homeobox Transcription Factor 1 Alpha (*LMX1A*), FEV Transcription Factor (*FEV*), Sodium Voltage-Gated Channel Alpha Subunit 3 (*SCN3A*), Transient Receptor Potential Cation Channel Subfamily M Member 3 (*TRPM3*), Secretogranin II (*SCG2*), Neurotensin (*NTS*), Peptide YY (*PYY*), Cholecystokinin (*CCK*), and *ADGRG4*. Subtypes were defined using UMAP embeddings to resolve spatially distinct cell subpopulations. Cells were partitioned into groups based on UMAP value distributions using empirically defined cutoffs in relation to canonical marker expression, and the resulting groups were used for downstream transcriptional identity analyses. Genes upregulated in each subtype were then used to define serotonergic and non-serotonergic transcriptional identity signatures. These signatures were quantified in all EC and SI-NET cells using Seurat’s AddModuleScore function, generating serotonergic and non-serotonergic module scores per cell. The difference between these scores defined a continuous transcriptional identity-axis value representing each cell’s relative transcriptional alignment with serotonergic versus non-serotonergic EC identity.

Group-wise comparison of the serotonergic-axis score distributions across serotonergic ECs, non-serotonergic ECs, and SI-NET cells were performed. Summary statistics (mean, median, and standard deviation) were calculated and effect sizes (Cohen’s d) were estimated using R package Effsize v0.8.1 [27]. Bootstrapped resampling (1000 iterations, equal group sizes) was used to estimate confidence intervals and assess the robustness of similarity estimates.

Significant genes were analyzed using interaction networks in STRINGdb v12.0 [28] and visualized in Cytoscape v3.10.3 [29]. Functional enrichment analysis was performed using g:Profiler (Ensembl 113) [30] to identify overrepresented biological processes and pathways.

## 3. Results

### 3.1. Cell Type Identification and Gene Expression Differences

**Figure 1:**
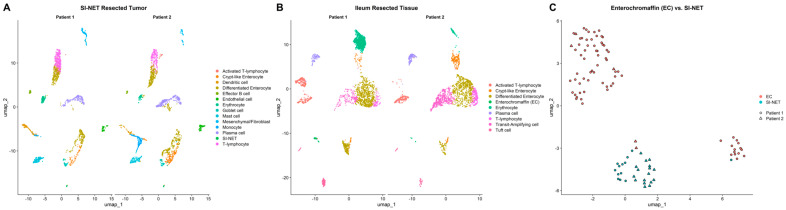
UMAP plots of cells from the discovery dataset. (**A**) SI-NET Resected Tumor cells (*n* = 3544). (**B**) Ileum Resected Tissue cells (*n* = 5419). (**C**) Enterochromaffin (EC) vs. SI-NET cells (*n* = 76 vs. 30). Identified cell types include activated T-lymphocytes, T-lymphocytes, differentiated enterocytes, dendritic cells, crypt-like enterocytes, transit-amplifying cells, erythrocytes, monocytes, plasma cells, effector B cells, mast cells, mesenchymal/fibroblasts, endothelial cells, goblet cells, tuft cells, enterochromaffin (EC) cells, and SI-NET cells.

We performed single-cell RNA sequencing on tumor tissue and adjacent normal intestinal mucosa, yielding 8963 cells. Unsupervised clustering and marker gene analysis identified distinct cell populations, and differential expression analysis between EC cells (*n* = 76) and SI-NET cells (*n* = 30) revealed 767 significant differentially expressed genes (DEGs; *p*.adj ≤ 0.05). Analysis of the external validation dataset, comprising EC (*n* = 87) and SI-NET (*n* = 660) isolated cells, identified 1921 significant DEGs (*p*.adj ≤ 0.05; Table S2) of which 370 overlapped with the discovery dataset and were retained for further analysis, including 272 upregulated and 98 downregulated genes (Table S3). UMAP visualizations demonstrated clear separation between tumor and normal cell clusters, illustrating distinct population identities (Figure 1).

### 3.2. Gene Interaction Network

**Figure 2:**
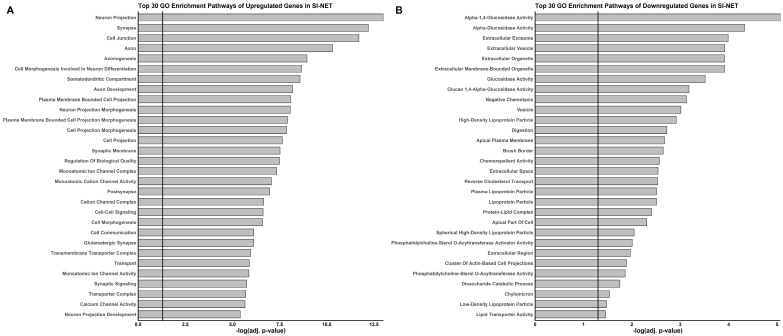
Top 30 Gene ontology (GO) analysis of the 75 SI-NET genes that are upregulated (**A**) or downregulated (**B**). The black line represents the significance threshold (adj. *p*-value = 0.05).

Clustering of the 370 significantly overlapping genes at a minimum interaction score of 0.9 identified 75 differentially expressed genes in tumor cells, comprising 47 upregulated and 28 downregulated genes (Table S4). Gene enrichment analysis showed that upregulated genes were associated with ion transport, synaptic signaling, and cytoskeletal organization, as well as axonogenesis, intercellular communication, and structural organization. Downregulated genes were enriched for enzymatic activity, lipid transport, and chemorepellent signaling, corresponding to reductions in digestion, cholesterol transport, and membrane-associated secretion and absorption. The top 30 Gene Ontology (GO) terms for both up- and downregulated genes are shown in Figure 2.

### 3.3. SI-NET and Enterochromaffin Cell Identity

**Figure 3:**
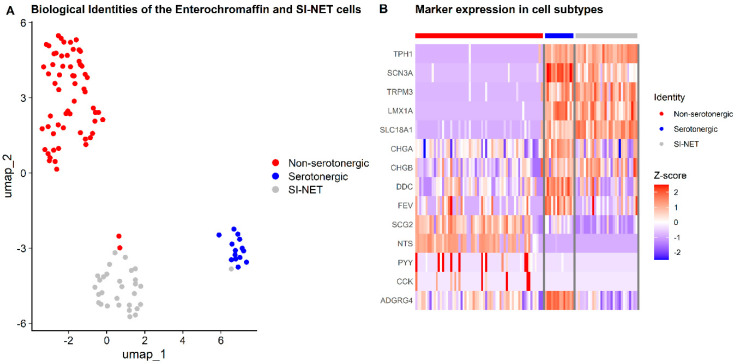
Subtyping of enterochromaffin (EC) and SI-NET cells using enteroendocrine markers. (**A**) UMAP visualization of EC cells from healthy ileum and SI-NETs, colored by biological identity: Non-serotonergic (red), Serotonergic (blue) and SI-NET (gray). (**B**) Heatmap showing expression of canonical enteroendocrine markers that distinguish serotonergic EC and non-serotonergic EC subtypes, alongside SI-NET cells. Markers shown: *TPH1*, *DDC*, *SLC18A1*, *CHGA*, *CHGB*, *LMX1A*, *FEV*, *SCN3A*, *TRPM3*, *SCG2*, *NTS*, *PYY*, *CCK*, *ADGRG4*.

To further determine subtypes, we attempted to infer the underlying genotypes using conventional single-cell CNV inference approaches; however, no conclusions could be estimated, most likely due to the limited number of analyzed cells. As shown in Figure 1C, EC cells are segregated into two distinct clusters, reflecting population heterogeneity. To further investigate subgroup differences, tumor cells were compared separately with each EC cluster (Figure 3A) using EC-specific marker profiles. The top cluster lacked serotonin-related markers, whereas the bottom cluster expressed high levels of serotonin biosynthesis genes (*TPH1*, *SLC18A1*, and others) (Figure 3B). Subgroup differentiation of non-serotonergic and serotonergic EC cells was also conducted in the external validation dataset (Figure S1). Among membrane proteins previously reported as neuroendocrine-specific, *SLC18A1* and *ADGRG4* exhibited distinct expression patterns between populations. *SLC18A1* was robustly expressed in both SI-NET and serotonergic cells but showed minimal expression in the non-serotonergic cluster. In contrast, *ADGRG4* reached maximal expression in serotonergic ECs while being more modestly expressed in both SI-NET and non-serotonergic cells (Figure 3B). Differential expression analysis identified 122 significantly different genes (*p*.adj ≤ 0.05) between SI-NET cells and serotonergic ECs, compared with 658 (*p*.adj ≤ 0.05) between SI-NET cells and non-serotonergic ECs (Table S5).

**Figure 4:**
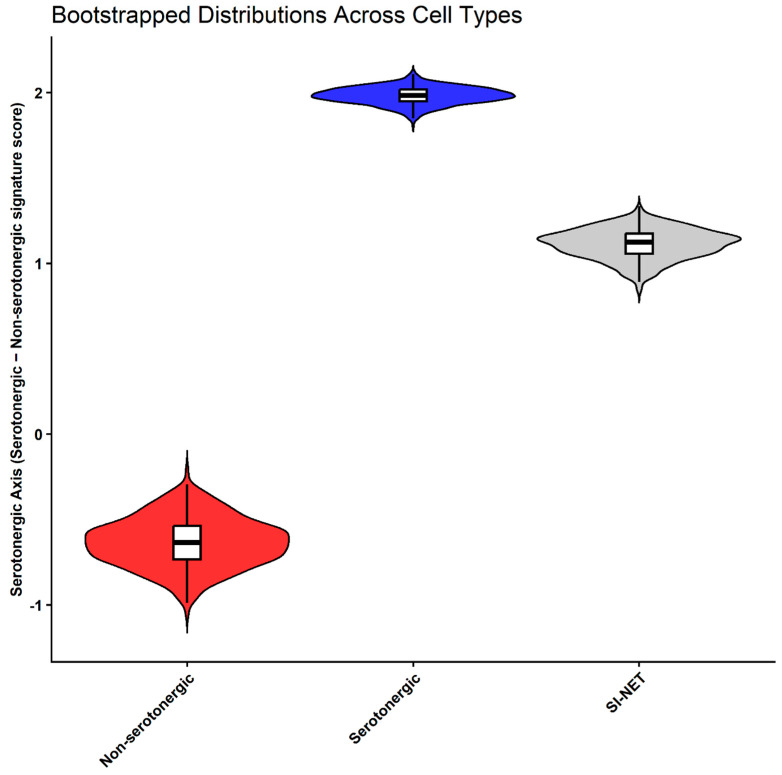
Bootstrapped distributions of serotonergic-axis scores for each cell identity. Plot depict the full score distribution with median and interquartile ranges indicated. The plot display Non-serotonergic scores in red, Serotonergic scores in blue, and SI-NET scores in gray.

Consistent with the smaller number of differentially expressed genes, transcriptional similarity analyses based on serotonergic and non-serotonergic gene signatures indicated that our patient SI-NET cells are positioned closer to their serotonergic ECs (Figure 4). Along the transcriptional identity gradient, defined by the difference in module scores between serotonergic and non-serotonergic gene sets, serotonergic ECs displayed the highest values (mean = 1.98 ± 0.19, bootstrapped mean = 1.98, 95% CI = 1.89–2.07), non-serotonergic ECs the lowest (mean = −0.64 ± 0.55, bootstrapped mean = −0.634, 95% CI = −0.920 to −0.352), and SI-NETs an intermediate profile (mean = 1.11 ± 0.35, bootstrapped mean = 1.11, 95% CI = 0.94–1.28). Bootstrapping analyses confirmed that SI-NETs were further similar to serotonergic ECs (mean similarity = 0.67 ± 0.04, 95% CI = 0.58–0.74) and less similar to non-serotonergic ECs (mean similarity = 0.33 ± 0.04, 95% CI = 0.26–0.42), indicating stronger alignment with the serotonergic ECs (Figure 4). All pairwise group comparisons were significant (*p*.adj ≤ 1 × 10^−8^) with large effect sizes (Cohen’s d ≥ 2.7). SI-NETs were 0.87 units from serotonergic ECs and 1.75 units from non-serotonergic ECs, representing roughly a two-fold difference.

### 3.4. Candidate Genes with Reciprocal Gain-Loss Expression

We compared our 122 significantly expressed genes from the SI-NETs versus serotonergic EC analysis with the 1242 significant genes (*p*.adj ≤ 0.05) identified in the corresponding analysis in the external validation dataset (Figure S1; Table S6), resulting in an overlap of 98 significant genes (Table S7). Among these overlapping genes, eight displayed no detectable expression in SI-NET cells; however, all exhibited enteroendocrine-specific expression in the small intestine according to the Human Protein Atlas. Conversely, nineteen genes were expressed in SI-NET cells but were absent in serotonergic ECs. Of these, eight lacked enteroendocrine expression in the Human Protein Atlas, and one gene (*LINC02484*) could not be validated due to missing reference data (Table 1).

## 4. Discussion

Single-cell analyses of enterochromaffin (EC) cells have so far primarily focused on their roles in gastrointestinal physiology [31,32,33]. Within the field of small intestinal neuroendocrine tumors (SI-NETs), single-cell technologies have been applied to compare primary tumors with metastatic lesions [12] and to examine intertumoral diversity [34]. Recently, a large multi-omic profiling study of SI-NETs identified four molecular subgroups [35], underscoring a level of heterogeneity that cannot be captured in smaller cohorts such as ours. The subgroup with the poorest prognosis was characterized by a mesenchymal gene expression signature and abundant cancer-associated fibroblasts, suggesting an intriguing avenue for future single-cell studies.

To date, investigations directly comparing EC cells with SI-NETs have primarily relied on bulk-tissue methodologies [36], limiting cellular resolution and specificity. Here, we present a single-cell RNA sequencing study that directly compares SI-NET cells with their cells of origin, the EC cells. To minimize false positives, we implemented a two-step validation strategy: first, by overlapping our findings with an external single-cell dataset, this approach identified 767 differentially expressed genes in the discovery dataset, approximately half of which overlapped with the external validation dataset. And second, by cross-referencing candidate genes against intestinal enteroendocrine cell expression with the Human Protein Atlas, resulting in genes with either gain or loss of expression in SI-NET cells.

Functional enrichment analysis indicates that our SI-NETs generally acquire neuronal-like signaling features while losing specific enteroendocrine characteristics. Rather than merely losing their enterochromaffin identity, our SI-NETs appear to undergo functional reprogramming. This process is characterized by both the loss of normal enteroendocrine functions and the acquisition of neuronal-like signaling properties, as supported by cross-dataset validation. These findings suggest a broader phenotypic shift toward a more neuronally active tumor state.

To further define patient specific characteristics, transcriptomic analysis of the EC population revealed two distinct subtypes, corresponding to serotonergic and non-serotonergic ECs (Figure 3A). The classification of these EC subtypes was based on serotonin related gene marker expression patterns, where serotonergic ECs and SI-NET cells shared overlapping expression of genes involved in serotonin synthesis and transport, whereas the non-serotonergic EC cluster lacked detectable serotonin biosynthetic activity (Figure 3B). Comparative profiling indicated that the SI-NET cells aligned more closely with the serotonergic subtype and the broader phenotypic shift from a secretory toward a neuronally active tumor state also appears to occur in the serotonergic EC relation.

The serotonergic-alignment pattern was also evident in the observed similarity scores and was reinforced by a bootstrapped distribution that corrected for sample-size imbalance, yielding a similarity of ~0.67 to serotonergic ECs along the transcriptional identity axis. The closer overall alignment with the serotonergic subtype suggests that this EC population most likely represents the cell of origin in our patients. This interpretation is further supported by elevated circulating 5-HIAA levels observed clinically, consistent with a serotonergic biochemical phenotype.

Consequently, comparisons with the non-serotonergic cluster may capture differences unrelated to tumor specificity, potentially obscuring biologically meaningful associations within the dataset. Consistent with the validation strategy applied in prior analyses, external dataset comparison confirmed that 98 genes corresponding to four out of five differentially expressed genes identified in the SI-NET versus serotonergic EC comparison were retained as overlapping in the external dataset. However, the limited number of cells analyzed restrains both the resolution and the general applicability of this observation. Larger single-cell datasets and independent patient cohorts will therefore be required to validate and further explore the proposed relationship and to determine whether serotonergic differentiation consistently underlies SI-NET development.

Given the limited number of cells analyzed, which constrains the generalizability of our conclusions, we focused on genes that were either lost or activated in our tumor cells using the Human Protein Atlas as a reference for enteroendocrine specificity. These alterations collectively define a loss-of-expression (LoE) or gain-of-expression (GoE) group (Table 1). For instance, SI-NETs lacked expression of the Notch ligand Delta-like ligand 1 (*DLL1*), consistent with impaired Notch-mediated differentiation. Additional LoE genes such as CEA Cell Adhesion Molecule 20 (*CEACAM20*), Polycystic Kidney and Hepatic Disease 1 Like 1 (*PKHD1L1*), Envoplakin (*EVPL*), Fatty Acid–Binding Protein 5 (*FABP5*), and Nephronectin (*NPNT*) are normally expressed in enteroendocrine cells and further underscore the loss of metabolic and adhesion functions that distinguish our SI-NETs from their cell of origin.

Conversely, the GoE group (Table 1) displayed enrichment for neuronal adhesion and signaling features. This group included neurotransmitter-related receptors: Glutamate Ionotropic Receptor AMPA Type Subunit 2 (*GRIA2*), Glycine Receptor Alpha 3 (*GLRA3*) and genes associated with synaptic stability: Contactin Associated Protein Family Member 5 (*CNTNAP5*), Catenin Alpha 2 (*CTNNA2*) suggesting alterations that may enhance intracellular signaling and tumor-microenvironment interactions. The identification of activated transcriptional regulators such as Transcriptional Repressor GATA Binding 1 (*TRPS1*) and Myelin Transcription Factor 1 Like (*MYT1L*) highlights potential molecular vulnerabilities that should be explored in greater detail in our patient population.

## 5. Conclusions

Based on the loss-gain expression, we hypothesize two potential therapeutic avenues with direct clinical application to our patients that warrant further investigation. First, restoring involved pathways such as Notch signaling may help restore growth control and limit tumor progression. Second, targeting aberrantly activated neuronal signaling networks could exploit tumor-specific vulnerabilities unique to our patient SI-NETs. From a clinical perspective, these strategies hold promise for developing more tailored and mechanism-based treatment in our patient cohort. Several candidate genes identified in this study could be prioritized for functional validation in preclinical models and further evaluated for relevance within stratified SI-NET patient populations (Table 1).

The primary limitation of this study is the small cohort size; the limited number of patients and cells reduces statistical power and increases susceptibility to donor-specific effects. This limitation is particularly relevant to interpretations suggesting patient-specific biology, such as the proposed serotonergic relationship. To enable stratification by key clinical parameters such as tumor grade, Ki-67 index, and disease stage, a more robust study design with an increase in patient samples is needed. In parallel, inclusion of multiple independent control donors for matched enterochromaffin comparison would be necessary to adequately capture the biological variability of enterochromaffin cells. Increasing both tumor and control cell numbers would improve statistical power, reduce donor bias, and allow for more reliable assessment of whether the identified transcriptional programs represent shared tumor features rather than patient-specific effects. Such a design would support statistically meaningful comparisons and strengthen conclusions regarding tumor biology to determine whether the identified genes function as tumor drivers or represent markers of cellular transformation.

## Figures and Tables

**Table 1:** Enteroendocrine expression–verified genes (*n* = 16) showing gain (GoE) or loss (LoE) of expression in SI-NET cells compared to serotonergic EC cells.

SI-NET LoE	SI-NET GoE
*BRINP3*	*CNTNAP5*
*CEACAM20*	*CTNNA2*
*DLL1*	*DACH2*
*EVPL*	*GLRA3*
*FABP5*	*GRIA2*
*NPNT*	*MYT1L*
*PKHD1L1*	*SYT14*
*PPP1R1C*	*TRPS1*

## Data Availability

We obtained single-cell transcriptome data for the external validation dataset from GSE125970 for the small intestine and GSE140312 for the primary small intestine neuroendocrine tumor. The raw data generated in this study has been deposited into the Federated European Genome-phenome Archive (FEGA) in Sweden (https://ega-archive.org). Data submitted to the archive is subject to controlled access, meaning that access will be granted only by following a formal application procedure in compliance with the General Data Protection Regulation (GDPR).

## Supplementary Materials

The following supporting information can be downloaded at https://www.mdpi.com/article/10.3390/cancers18030435/s1: Table S1: Cellmarkers; Table S2: Validationdataset; Table S3: DiscoveryValidationOverlap; Table S4; NetworkGeneNodes; Table S5: SINET_vs_Serotonergic_vs_Nonserotonergic; Table S6: External_SINET_vs_Serotonergic; Table S7: SINET_vs_Serotonergic_ExternalOverlap; Figure S1: Subtyping of external validation enterochromaffin (EC) and SI-NET cells using enteroendocrine markers.
