# Supplementary figures and images for: Single-Cell Comparison of Small Intestinal Neuroendocrine Tumors and Enterochromaffin Cells from Two Patients
Source: Cancers (Basel). 2026 Jan 29;18(3):435. doi: 10.3390/cancers18030435 (PMC12897003; doi:10.3390/cancers18030435)

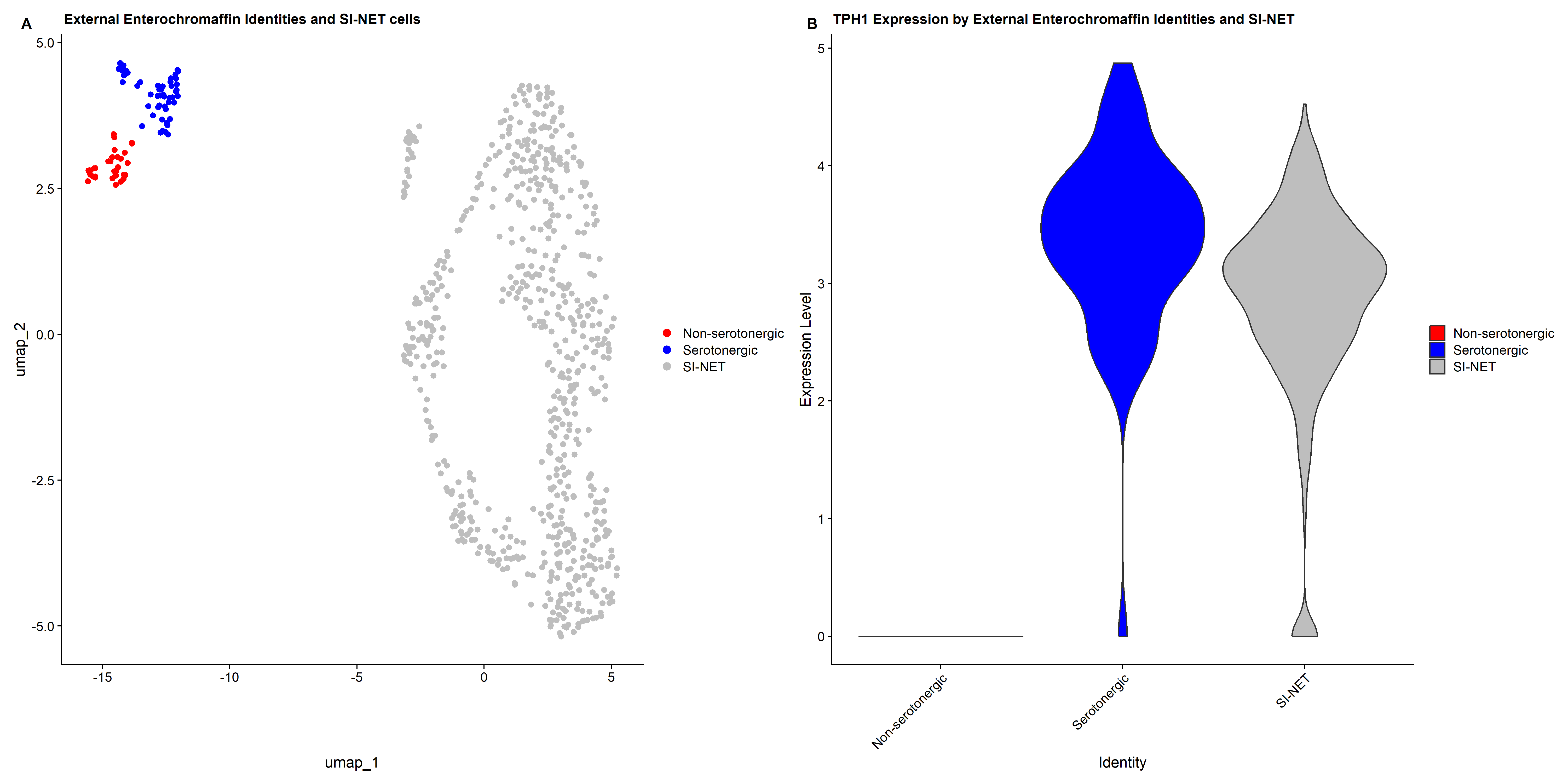

Supplement: Supplementary file 1 [file cancers-18-00435-s001.zip › FigureS1.tif]
